# The destination of Pacific Island health professional graduates from a New Zealand university

**DOI:** 10.1186/1478-4491-10-24

**Published:** 2012-08-20

**Authors:** Shiva M Nair, Prabal R Mishra, Pauline T Norris, Charlotte Paul

**Affiliations:** 1 Department of Pharmacology and Toxicology, University of Otago, Dunedin, New Zealand; 2 Department of Preventive and Social Medicine, University of Otago, Dunedin, New Zealand; 3 School of Pharmacy, University of Otago, Dunedin, New Zealand; 4 Department of Preventive and Social Medicine, University of Otago, PO Box 913, Dunedin, New Zealand

**Keywords:** Developing country, Health professional, Migration, Pacific Islands, Workforce

## Abstract

**Background:**

There is a shortage of health professionals in Pacific Island states and territories, and a need in New Zealand for Pacific health professionals to serve Pacific communities.

**Methods:**

A cross-sectional postal survey was conducted to investigate retention of Pacific graduates. All graduates of Pacific ethnicity or nationality from the University of Otago in the years 1994 to 2004 in medicine, dentistry, pharmacy, physiotherapy and medical laboratory science were included.

**Results:**

The response rate was 59% (75 out of 128). Only 7% of respondents were working in the Pacific Islands (12% of non-residents and 4% of New Zealand residents), though the proportion in the whole cohort could be up to 20%. One third intended to work in Pacific communities in New Zealand or the Pacific Islands in the future. Factors that would favour such an intention were an adequate income, job availability, and good working conditions.

**Conclusions:**

Retention of graduates in the Pacific Islands is poor and measures to improve retention are needed.

## Background

The globalisation of the labour market has exacerbated the health worker shortage in developing countries, as workers trained in those countries, or at their expense, are attracted to work in richer countries [1]. In the Pacific, the migration of Pacific Island health workers to New Zealand and Australia and beyond has been referred to as a “brain drain” [2]. Recent estimates of the health workforce in Pacific Island states and territories demonstrate how critical the shortages are in medicine, dentistry, pharmacy, nursing and midwifery [3,4]. For instance, most Pacific states have only around one to five doctors per 10 000 population; in comparison, New Zealand has 29 doctors per 10 000 population [3,4]. The level of infrastructure development and health-care expenditure varies among the 13 Island states, but among those states are Solomon Islands, Tuvalu, the Republic of Vanuatu, Kiribati and the Independent State of Samoa, all of which have been listed among the least developed countries in the world [5].

Through years of migration, a substantial Pacific Island community has evolved in New Zealand, making up 6.4% of the population, of whom 60% were born in New Zealand [6]. Most identify their heritage from the Cook Islands, Samoa, the Kingdom of Tonga and the Republic of Niue. Pacific residents in New Zealand have a worse health status than the total population [7], and increasing the Pacific health workforce is one of the ways of overcoming potential cultural and language barriers to health messages and health care [8].

Currently, there are two major medical training facilities in the Pacific: Fiji School of Medicine (Fiji National University), and University of Papua New Guinea, and also the recently formed Umanand Prasad School of Medicine (University of Fiji) and the Oceania University of Medicine (Samoa). The Universities of Auckland and Otago play a major part in the development of the Pacific workforce in New Zealand and to a certain extent in the Pacific. At Otago University, residents of Pacific descent are preferentially selected into health professional courses in medicine, dentistry, pharmacy, and physiotherapy. A number of non-New Zealand resident Pacific students are sponsored through New Zealand Agency for International Development (NZAID) or government scholarships, or pay privately, but little is known of their destination after graduation. We undertook a survey of both resident and non-resident graduates of Pacific ethnicity from health professional undergraduate courses at the University of Otago to examine the retention of these graduates in Pacific communities and factors influencing their choices of destination.

## Methods

A cross-sectional survey was undertaken of all graduates of medicine, dentistry, pharmacy, physiotherapy and medical laboratory science who were either of Pacific Island nationality or ethnicity from 1994 to 2004 from the University of Otago. Data supplied by the University of Otago was also used to determine the residency status of the sample and of the respondents.

Eligible participants were identified from the Division of Health Sciences records. Ethnicity was self-identified by the students during enrolment. A total of 134 graduates were identified. Current addresses were sought firstly through personal networks (35 identified, 28 replied), and secondly, through New Zealand registration authorities: Medical Council of New Zealand (21 identified as sole source, 11 replied), New Zealand Pharmacy Council (21 identified, 11 replied), Medical Laboratory Scientists Board and Physiotherapy Board of New Zealand. The Dental Council addresses are available on their website and the Medical Council released addresses; the other bodies declined to release addresses but agreed to forward letters. Thirdly, if addresses were still unavailable, an internet search using Google, and social networking sites such as Facebook was undertaken. Finally, if no recent address could be identified (12), the last known address was used. This latter resulted in no eligible responses.

A questionnaire, seeking information on current location, type of work and career intentions, was posted to graduates with an identified address during March to December 2008. In Samoa, three were hand-delivered. If no reply was received, a follow-up questionnaire was sent after three months.

Ethical approval for the study was obtained from the Department of Preventive and Social Medicine, University of Otago.

## Results

A total of 79 people responded to the survey, four respondents were excluded as they did not have a Pacific ethnicity or nationality. A further two non-respondents were excluded for the same reason. Hence the response rate from known eligible graduates was 75 out of 128 (59%) and 65% of those for whom a recent address was identified. Characteristics of respondents and non- respondents are shown in Table 1. More women than men responded. The response rate was highest for dentistry (74%) and lowest for medical laboratory science (33%). It was highest for graduates of Tongan ethnicity (89%) and lowest for graduates of Other Pacific ethnicity (29%); where the location of practice was known, the highest response rate was from New Zealand (71%) and the lowest from the Pacific (43%). Of the total sample, 37% were non-residents at the time of admission to University; of the respondents, 25 (33%) were non-residents.

**Table 1:** Characteristics of responders and non-responders

	**Responders (n = 75)**	**Non-responders (n = 53)**
Mean age	30.3	
Percentage of women	64%^a^	47%
Average years since graduation	7.5	8.3
**Course**		
Medicine	28 (57%)	21 (43%)
Pharmacy	23 (56%)	18 (44%)
Dentistry	14 (74%)	5 (26%)
Physiotherapy	7 (70%)	3 (30%)
Medical Laboratory Science	3 (33%)	6 (66%)
**Ethnicity**		
Cook Islands Maori	2 (40%)	3 (60%)
Fijian	9 (75%)	3 (25%)
Fijian Indian	30 (63%)	18 (37%)
Samoan	15 (71%)	6 (29%)
Tongan	8 (89%)	1 (11%)
Multiple Pacific	5 (42%)	7 (58%)
Other Pacific^b^	6 (29%)	15 (71%)
**Main location of practise**		
New Zealand	48 (71%)	20 (29%)
Pacific Islands	5 (43%)	7 (57%)
Elsewhere	17 (63%)	10 (37%)
Unknown	0 (0%)	16 (100%)
Not practising	5	Unknown

^a^Row percentages; ^b^Melanesian, Vanuatuan, Niuean, Tokelauan, not stated.

Table 2 shows the workforce characteristics of respondents according to profession. As expected of recent graduates, the mean age was between 28 and 31 years and the mean years since graduation was seven to eight years. All except five people were still practising in their original profession. The proportions that were women ranged from 50% for dentistry to 100% for medical laboratory science. Of those with a medical qualification, Samoans were the largest ethnic group (32%), and for dentistry and pharmacy, Fijian Indians were the largest group (43% and 83% respectively). Postgraduate qualifications had been gained by 54% in medicine and 39% in pharmacy. Other professions had rates around 30%. Most people intended to undertake further postgraduate work, though fewer dentists or pharmacists intended to. The location of practice was in the Pacific Islands for only five respondents (all women); two in medicine, two in pharmacy, and one in physiotherapy. Another respondent, a dentist, was working among an Aboriginal community in Australia. A further 11 respondents said they were practising in Pacific Island communities in New Zealand, so that in all, 16 (21%) of respondents were practicing in Pacific communities. A further 21 respondents stated that they were working in areas with large Pacific communities, e.g. in South Auckland; six in medicine, one in dentistry, and 14 in pharmacy. Hence, just over half of all graduates were working with Pacific communities or in areas with large Pacific populations. Notably, 40% of both medical graduates and pharmacy graduates intended to work in Pacific communities at some time in the future; the proportions were lower for other groups. Overall, 17 (23% of respondents) were working elsewhere overseas, most in Australia.

**Table 2:** Workforce characteristics of responders according to profession

**Characteristic**	**Medicine**	**Dentistry**	**Pharmacy**	**Physiotherapy**	**Medical laboratory sciences**
N	28	14	23	7	3
Age (mean and range)	31 (26–43)	30 (26–34)	30 (25–35)	28 (25–34)	31 (29–34)
Number of women (%)	16 (57%)	7 (50%)	16 (70%)	6 (86%)	3 (100%)
Mean years since graduation (range)	7.3 (4–14)	7.3 (4–11)	7.8 (4–13)	7.0 (4–13)	7.7 (4–10)
Still practising as original health professional degree	28	13	20	7	2
**Ethnicity**					
Cook Islands Maori	1	0	0	1	0
Fijian	8	1	0	0	0
Fijian Indian	2	5	19	3	1
Samoan	9	2	1	2	1
Tongan	4	3	0	0	1
Multiple Pacific	1	2	1	1	0
Other Pacific	3	1	2	0	0
**Postgraduate status**					
Done postgraduate qualification	15	4	9	2	1
Intending to do postgraduate qualification	25	7	18	5	2
Not done nor intending to do postgraduate qualification	0	6	6	0	0
**Main location of practice**					
Pacific Island community in NZ	5	1	2	3	0
General community in NZ	15	9	11	0	2
Pacific Islands	2	0	2	1	0
Elsewhere	6	3	5	3	0
Number ever worked in Pacific Island community in NZ	13	3	5	2	1
Number ever worked in the Pacific	10	5	12	3	0
Intending to work in Pacific Island community/Pacific Islands if not currently	11	2	10	1	1
**Mentor status**					
Currently has a mentor	4	6	10	2	0
Mentor is a Pacific Islander	3	0	3	1	-
Mentor is helpful	4	6	10	2	-
Mentor influenced decisions on where to practise	4	1	3	1	-

NZ: New Zealand.

Although only five respondents were currently working in the Pacific, 30 (40%) had worked there in the past, for a mean of 2 to 4 years. Similarly, 24 (32%) had worked in Pacific communities in New Zealand in the past.

Overall, 22 (30%) of respondents currently had a mentor, all of whom were reported to be helpful, and seven of whom (32%) were of Pacific ethnicity. For nine respondents the mentor had influenced decisions on where to practice, though in one case that influence was towards working in the general community.

A comparison of non-New Zealand resident and resident respondents according to selected workforce characteristics is shown in Table 3. Although the numbers are too small for robust analysis, a higher proportion of non-residents studied pharmacy and a lower proportion studied dentistry; a lower proportion of non-residents completed a postgraduate qualification and more did not intend to complete one in the future. A higher proportion (12%) of non-residents were practicing in the Pacific Islands than residents (4%), and 56% of non-residents had worked in the Pacific Islands in the past compared with 32% of residents. More non-residents (40%) intended to work in the Pacific community in New Zealand or the Pacific in the future, compared to 30% of residents.

**Table 3:** Selected workforce characteristics according to New Zealand resident status

**Characteristic**	**Non-resident (N = 25)**	**Resident (N = 50)**
**Course**		
Medicine	9 (36%)^a^	19 (38%)
Dentistry	3 (12%)	11 (22%)
Pharmacy	10 (40%)	13 (26%)
Physiotherapy	2 (8%)	5 (10%)
Medical laboratory science	1 (4%)	2 (4%)
Completed postgraduate qualification	6 (24%)	25 (50%)
No postgraduate qualification and not intending to do	6 (24%)	6 (12%)
**Main location of practice**		
Pacific Island community in NZ	4 (16%)	7 (14%)
General Community in NZ	15 (60%)	22 (44%)
Pacific Islands	3 (12%)	2 (4%)
Elsewhere	3 (12%)	14 (28%)
Ever worked in the Pacific Islands	14 (56%)	16 (32%)
Intending to work in Pacific Islands community in NZ or Pacific Islands if not currently	10 (40%)	15 (30%)

^a^Column percentages. NZ: New Zealand.

Those working in the Pacific were asked what factors influenced them in the choice of place of work. Two were bonded to work there, one mentioned the shortage of doctors and family ties, one mentioned job opportunities and the remaining woman stated simply that this was where she was from.

Those not currently in the Pacific or in Pacific communities in New Zealand were asked what factors would favour or hinder them working there. The factors that would favour an intention to work with Pacific communities are shown in Table 4. Some answers related to the current situation and some looked to situations in the future. An adequate income was most commonly mentioned, followed by job availability and appropriate facilities and opportunities for further training. But next most common was to be near family, and seeing a need in Pacific communities, being able to meet that need, to give something back, and to feel appreciated. This went along with job satisfaction, understanding the culture and language and Pacific health issues, and lifestyle. It would encourage people to work in these communities if there was more support and guidance, if there was scope for clinical and research projects, and if there was more active recruitment including preferential selection of Pacific doctors to hospitals with high Pacific populations in New Zealand, and short term placements to gain experience. One respondent mentioned a need for Fijian Indians to be regarded as part of the Pacific health workforce.

**Table 4:** Factors that would favour an intention to work in a Pacific community (New Zealand or Pacific Island) in the future

	**Number of respondents mentioning**^**a**^
Adequate income	12
Job availability/opportunities/appropriate facilities/working conditions/further training opportunities	10
Near family, family life	8
Seeing a need in the community/a sense of helping/giving something back/making a difference/being appreciated	8
Job satisfaction	5
Lifestyle	4
Support and guidance from Pacific organisations, professional community and senior health professionals	3
Understanding culture, language and Pacific health issues	3
Active recruitment/short term placement	3
Less busy	2
Scope for clinical projects/research	2

^a^Some respondents mentioned more than one; not all respondents gave a comment.

Table 5 shows factors that currently hinder working with Pacific communities. Poor pay was mentioned most often, followed by cultural barriers to working with Pacific people, and then by a lack of facilities and infrastructure for specialist practice and lack of advanced training opportunities. Lack of job opportunities, not knowing about job opportunities, family commitments, student loan debt, lack of support or encouragement, political instability and local political difficulties, and isolation from peers were all mentioned by two or more people.

**Table 5:** Factors that would hinder an intention to work in a Pacific community (New Zealand or Pacific Island) in the future

	**Number of respondents mentioning**^**a**^
Poor pay	14
Cultural barriers (language, non-compliance, low socioeconomic status, inability of patients to pay)	7
Lack of facilities and infrastructure for specialist practice	6
Lack of advanced training opportunities	6
Family commitments	5
Lack of job opportunities	5
Student loan debt	3
Lack of support/encouragement	3
Political instability and local politics	3
Not knowing of need or job opportunities	3
Isolation from peers	2

^a^Some respondents mentioned more than one; not all respondents gave a comment.

Lastly, respondents commented on whether there was anything during their training at the University of Otago which influenced their decision to work with Pacific communities, either for or against. Most described no influence and three described factors against: no specific health sciences mentor, no offers or positions, and student loan debt repayment. For the seven who described positive influences, these were knowledge of health problems for Pacific people, the need for people to work in the Pacific, and a Pacific mentor.

## Discussion

Among a cohort of Pacific health professional graduates from one University, by around seven years after graduation only 7% of respondents were working in Pacific Island states, though 20% were working among Pacific communities in New Zealand. Nevertheless, 40% had worked in the Pacific Islands in the past and up to this proportion intended to work there in the future. The main factors that would favour such an intention were an adequate income, job availability, and good working conditions. A wish to give something back and presence of family were also important motivators.

There were some differences between non-residents who came from Pacific Island states to study in New Zealand compared with residents of Pacific ethnicity, especially in the higher proportion working in the Pacific (12% versus 4% of residents), and in the proportion intending to work in Pacific communities in the future (40% versus 30% of residents). Nevertheless there were similarities in that a substantial proportion of both groups had ties to the Pacific, as shown by the fact they had worked there in the past, hence the groups were combined for the main analyses.

Limitations of the study include the low response rate from health professionals working in the Pacific, and from people of “other Pacific” ethnicity. Non-responders were more likely to be known to be working in a Pacific Island state, and for the 16 where location could not be ascertained, it is plausible that a disproportionate number might have resided outside New Zealand. Hence, of all graduates sampled, 12 out of 128 (9%) were known to be working in the Pacific, but conceivably, up to 28 out of 128 (22%) may have done so – assuming all those of unknown location were in the Pacific. In addition, information pertains largely to graduates from Fiji, Samoa and Tonga, and the results cannot be extrapolated to other Pacific Island states. To tease out the precise meanings of the factors investigated, other in-depth methods are required.

The problem of the migration of skilled health professionals from poorer countries to rich countries [1] is not so clear-cut in this case. New Zealand has a large Pacific population itself and the Ministry of Health is keen to increase the proportion of the health workforce that is of Pacific ethnicity. However, some of the countries in the Pacific from which these students came are amongst the least developed in the world. A total of 37% of all the graduates sampled (and 33% of responders) came from the Pacific as international students, i.e. to study on either New Zealand Development Aid or government scholarships or with private funding. Hence the Pacific Island governments, individual families or NZAID are paying for these students to be educated and yet the home country is losing their skills and expertise.

The ratio of health professionals to population in these Pacific Island states is many times worse than in New Zealand [3]. New Zealand is just one of the countries that educates health professionals for the Pacific, so the responsibility for improving the situation is shared. Short-term return to the Pacific of graduates seems common and this may partly be due to bonding systems. A period following this of specialization or further training in New Zealand or another developed country may be highly appropriate, though this could also happen at regional centres such as Fiji. The experience in Fiji shows that it is also vital to establish a predictable career progression path to encourage completion of training [9]. For those doing advanced training in New Zealand, the important point comes when it is hoped that these professionals would move back to the Pacific. Thus the comments on factors that would favour or hinder such a move at this time after graduation are especially important.

Recommended national strategies to stem the migration of health workers out of poor countries to fill skills shortages in rich countries are highly relevant [10]. These reflect the issues mentioned by respondents. For the poor countries, these include improving working conditions for health professionals, requiring publicly funded trainees to commit to a specified period of national service, and pursuing policies that emphasise the development of science and technology research. The rich countries that receive these migrant health workers trained at the expense of their home countries should pay compensation to the source country. In New Zealand, experience has shown that the most successful strategy for retention of doctors in the Pacific has been by measures aimed at mentoring and supporting graduates over the long term to complete advanced training and return to positions in their Island states (personal communication, Mr Tearikivao (Kiki) Maoate).

The Universities that provide health professional education need to continue and strengthen teaching about Pacific health, stress the need for Pacific health professionals to work in the Pacific and with Pacific communities in New Zealand, and to provide Pacific mentors. Health professional teachers should encourage students to examine their personal motivation to serve their own communities [11]. The undergraduate years are an ideal time to foster this interest and make vital connections for the future. Universities, other tertiary institutions and health-services providers in New Zealand could also provide opportunities for training and involvement in research for health professionals working in the Pacific, and indeed partner with Pacific states to share health resources. In particular, New Zealand academic institutions could provide support for in-country training in Fiji and Samoa through such methods as web-based teaching (webinars), providing second opinions in difficult cases and enabling exchanges of senior health professionals in both directions.

## Conclusions

There is poor retention of Pacific health professional graduates from New Zealand in Pacific Island states, though lower response rates from those known to be working in the Pacific makes precise determination difficult. Strategies to encourage Pacific graduates to return to Pacific Island states and to work among Pacific communities in New Zealand are required. Further in-depth investigation of models that are being used successfully is needed so that this information can be shared across the Pacific.

## Competing interests

The authors declare that they have no competing interests.

## Authors’ contributions

SN and PM conceived the project, designed the study, and undertook the data collection and analysis. CP and PN supervised the design and conduct of the study and contributed to the interpretation. SN wrote the first draft of the paper and CP, PN and PM revised it for important intellectual content. CP is the guarantor. All authors read and approved the final manuscript.
